# P2X7 Receptor Activation Impairs Exogenous MHC Class I Oligopeptides Presentation in Antigen Presenting Cells

**DOI:** 10.1371/journal.pone.0070577

**Published:** 2013-08-05

**Authors:** Alberto Baroja-Mazo, Maria Barberà-Cremades, Pablo Pelegrín

**Affiliations:** Unidad de Inflamación y Cirugía Experimental, Centro de Investigación Biomédica en Red en el Área temática de Enfermedades Hepáticas y Digestivas (CIBERehd), Instituto Murciano de Investigación Biosanitaria (IMIB), Hospital Universitario Virgen de la Arrixaca - Fundación Formación Investigación Sanitaria Región Murcia (FFIS), Murcia, Spain; University Hospital Freiburg, Germany

## Abstract

Major histocompatibility complex class I (MHC I) on antigen presenting cells (APCs) is a potent molecule to activate CD8^+^ T cells and initiate immunity. P2X7 receptors (P2X7Rs) are present on the plasma membrane of APCs to sense the extracellular danger signal adenosine-5′-triphosphate (ATP). P2X7R activates the inflammasome and the release of IL-1β in macrophages and other immune cells to initiate the inflammatory response. Here we show that P2X7R stimulation by ATP in APCs decreased the amount of MHC I at the plasma membrane. Specific antagonism or genetic ablation of P2X7R inhibited the effects of ATP on levels of cellular MHC I. Furthermore, P2X7R stimulation was able to inhibit activation of CD8^+^ T cells via specific MHC I-oligopeptide complexes. Our study suggests that P2X7R activation on APCs is a novel inhibitor of adaptive CD8^+^ T cell immunity.

## Introduction

Adaptive immunity requires the activation of T lymphocytes by antigen presenting cells (APCs), which present antigen bound to major histocompatibility complex (MHC) molecules. Several cell types are responsible for antigen presentation to T cells, including macrophages, B-lymphocytes, dendritic cells, and Langerhans cells. Macrophages are important APCs, since they are ubiquitously localized through the body and initiate the immune response against pathogen by the production of cytokines and by their phagocytic, cytotoxic, and antigen-presenting capabilities [1]. The ability of macrophages to present antigens is crucial for the correct immune function, when it is compromise there is an increase risk for morbidity and mortality due to infection [2].

Intracellular antigens are presented by MHC class I (MHC I) molecules and activate cytotoxic CD8^+^ T cells, meanwhile extracellular antigens are presented by MHC class II (MHC II) molecules to activate CD4^+^ T helper cells [3], [4]. However, a mechanism termed cross-presentation permits some extracellular antigen to stimulate CD8^+^ T cells via the MHC I pathway of APCs [5], [6]. MHC I molecules are in a continuum recycling through a complex endosome pool and new MHC I molecules could traffic from the ER to the cell surface following the classical secretory pathway through the Golgi complex [7] or using an alternative route via the recycling endosome pool [8]. MHC I molecules that do not fold properly or which lack antigen peptide, are retro-translocated into the cytosol and degraded by the proteasome [9].

Extracellular nucleotides are emerging as potent modulators of the immune response, in particular high levels of extracellular adenosine-5′-triphosphate (ATP) is considered a danger signal associated *in vivo* with different inflammatory conditions [10], [11], [12], [13]. P2X7Rs are expressed in APCs and recognize high amounts of extracellular ATP. P2X7R is the most potent plasma membrane receptor responsible for the activation of the inflammasome, the release of pro-inflammatory cytokines of the IL-1 family, the production of eicosanoids, the increase of reactive oxygen species and the release of proteases to the extracellular space [10], [14], [15], [16]. Also P2X7R induces the shedding of different surface receptors, including MHC class II (MHC II) protein and CD62 ligand [17]. Experiments performed in P2X7R-deficient mice and with selective drug-like P2X7R antagonists have demonstrated a role for P2X7R in the progression of rheumatoid arthritis, lung inflammation, graft-versus-host disease, irritable bowel syndrome, fever, contact hypersensitivity, and inflammatory and neuropathic pain [11], [12], [13], [18], [19], [20], [21]. Therefore, P2X7R is a promising therapeutic target in the management of inflammation and pain, as witnessed by the large number of selective P2X7R antagonists developed by several drug companies and currently under clinical trials [20], [22].

In this study we investigated the specific role of P2X7R in APCs MHC I surface expression and we found that P2X7R stimulation not only induced a reduction of MHC I levels, but also impaired MHC I activation of CD8^+^ T cells. By using pharmacological and genetic tools we demonstrated that P2X7R stimulation decreased antigen presentation by MHC I in APCs, impairing an appropriate adaptive CD8^+^ T-response.

## Materials and Methods

### Animals

Mice were maintained in a pathogen-free, humidity- and temperature-controlled environment with 12 h light-dark cycles and free access to food and drinking water. All animal used in this work was in accordance with the Spanish national (RD 1201/2005 and Law 32/2007) and EU (86/609/EEC and 2010/63/EU) legislation. According to legislation cited above, local ethics committee review or approval is not needed, since as explained in the methods mice were euthanized by CO_2_ inhalation and used to obtain bone marrow or spleen; no procedure was undertaken which compromised animal welfare.

C57 BL/6 (wild type, WT) mice were purchased from Harlan. P2X7R-deficient mice (P2X7R^−/−^) were purchased from Jackson [23] and OT-Ix*rag-1^−/−^* mice (OT-I) were kindly provided by Dr. D. Sancho. Both mice were in C57 BL/6 background. For all experiments, males and females mice between 8–10 weeks of age bred under SPF conditions were used to obtain the bone marrow or spleen cells.

### Reagents and Buffers

The restricting K^b^ class I peptide OVA 257–264 (SIINFEKL) (OVA_257–264_) was from AnaSpec and the fluorescein isothiocyanate-conjugated OVA 257–264 peptide (OVA_257–264_-FITC) was synthesized by GenScript. PE conjugated anti-mouse MHC I(H-2K^b^)pOVA 257–264 and APC conjugated anti-mouse MHC-I (H-2k^b^) were from eBioscience, mouse seroblock FcR antibody from AbD Serotec, AlexaFluor 488 conjugated anti-mouse F4/80 from Caltag Laboratories and PE conjugated anti-mouse IFNγ from BD Bioscience. *Escherichia coli* LPS serotype 055:B5, ATP, MG132, etoposide, apyrase, pan-caspase inhibitor (Q-VD-OPh) and Brefeldin A were purchased from Sigma-Aldrich. Recombinant mouse IL-4 was from BD; caspase-3 inhibitor I (Ac-DEVD-CHO), GM6001 and E64 protease inhibitor from Calbiochem. P2X7R antagonists A438079 or A740003, the ecto-NTPDase inhibitor sodium polyoxotungstate (POM1), the ecto-5′-nucleotidase inhibitor adenosine 5′-(α,β-ethylene)diphosphate (APCP) and *N*,*N*,*N*',*N*'-*Tetrakis*(2-pyridylmethyl)ethylenediamine (TPEN) from Tocris. The composition of the physiological buffer used in the experiments (Et) was (in mM): NaCl 147, HEPES 10, D-glucose 13, KCl 2, CaCl_2_ 2 and MgCl_2_ 1; pH 7.4. In some experiments, an Et-buffer containing no added CaCl_2_ and supplemented with 1 mM EGTA (Et-EGTA buffer) was used.

### Preparation of Mouse Bone Marrow-derived Macrophages, Dendritic Cells and Splenocytes

Bone marrow-derived macrophages (BMDMs) were obtained from leg bones of mice euthanized by CO_2_ inhalation. Femurs and tibia were removed, the bone marrow was flushed out and resuspended in DMEM (Lonza) supplemented with 25% of L929 medium, 15% fetal calf serum (FCS, Invitrogen), 100 U/ml penicillin/streptomycin (Lonza), and 1% L-glutamine (Lonza), plated onto 150-mm dishes, and cultured at 37°C in the presence of 5% CO_2_. After 7 days, the resulting BMDMs were detached with cold PBS, replated into 96-, 24-, 12- or 6-well plates at a confluence of 0.42×10^6^ cells/cm^2^, and used the following day. The macrophage purity of these preparations was usually higher than 90% as routinely checked by flow cytometry with antigen specific F4/80 staining. Macrophage differentiation to phenotype M1 or M2 was performed as described before [24]. Bone marrow-derived dendritic cells (DCs) were obtained as BMDMs, but culturing bone marrow cells without L929 supplementation, but with 20 ng/ml of recombinant murine GM-CSF (PreProtech) for 6 days, and then with 500 U/ml of recombinant murine TNFα (Sigma-Aldrich) for two days. Splenocytes from OT-I mice were obtained by mechanical spleen disaggregation in a gentleMACS™ Dissociator (Miltenyi Biotech).

### Membrane MHC I Quantification

BMDM in 12-well plates were washed and incubated 30 min at 37°C in Et-buffer with 5 mM of ATP, in presence or absence of P2X7R antagonist A438079. After the incubation, cells were washed and medium was replaced with Et and incubated 2 hours more at 37°C in the presence of 5% CO_2_. Other wells were treated with 5 µg/ml of Brefeldin A during all the process. To stain surface MHC I (H-2K^b^), cells were washed and detached using cold PBS with 2 mM EDTA. Cells were first incubated with mouse seroblock FcR and then stained with anti-MHC I (H-2K^b^) APC conjugated for 30 min at 4°C. Finally, cells were washed and fixed with 4% *p*-formaldehyde (PFA) in PBS. Other samples were used to determine total MHC I by intracellular staining. For these experiments, fixed cells were permeabilized with Perm/Wash buffer (BD Biosciences) for 15 min at 4°C and then stained with anti-MHC I (H-2K^b^) APC. All samples were subjected to flow cytometry analysis using a BD FACSCanto flow cytometer (BD) and FACSDiva software (BD) by gating for BMDM cells based on FSC *versus* SSC parameters.

### Detection of MHC I-OVA_257–264_ Complexes

BMDM in 12-well plates were washed and incubated from 5 to 30 min at 37°C in Et-buffer with different concentrations of ATP. When indicated in the text, different inhibitors were added together with ATP. After the incubation, cells were washed and medium was replaced with Et containing 1.5 µg/ml of OVA_257–264_ and cells were incubated for further 2 h at 37°C in the presence of 5% CO_2_. To stain surface OVA_257–264_/K^b^ cells were washed, detached and stained as above using anti-MHC I(H-2K^b^)pOVA_257–264_ PE. Flow cytometry analysis was carried out in a BD FACSCanto flow cytometer (BD) and FACSDiva software (BD) by gating for BMDM cells based on FSC *versus* SSC parameters.

### CD8^+^ T cell Activation Assay

BMDM in 96-well plates were washed and incubated 30 min at 37°C in Et-buffer with 5 mM ATP followed by OVA_257–264_ peptide as explained above. After incubation with OVA_257–264_ peptide, BMDMs were co-incubated with 1×10^5^ OT-I splenocytes for 6 h in the presence of 5 µg/ml of Brefeldin A at 37°C with 5% CO_2_. Splenocytes were then collected, washed, fixed with 4% PFA and permeabilized with Perm/Wash buffer (BD Biosciences) for 15 min at 4°C. Splenocytes were stained with PE conjugated anti-IFNγ for 30 min at 4°C and analyzed by flow cytometry as above, by gating OT-I splenocytes based on FSC *versus* SSC parameters.

### OVA_257–264_ Uptake

BMDM cultured in 12 well plates were incubated 2 h with 5 µg/ml brefeldin A. Then cells were treated or not with ATP 5 mM for 30 min and incubated 45 min with 5 µg/ml OVA_257–264_-FITC at 37°C in the presence of brefeldin A. BMDM were gated based on FSC *versus* SSC parameters and analyzed by flow cytometry to determine fluorescent peptide uptake using a BD FACSCanto flow cytometer (BD). Similarly, BMDM cultured on poly-L-lysine coverslips were treated as above. After peptide incubation, coverslips were fixed with 4% PFA, washed and inverted onto microscope slides with Fluoroshield containing DAPI (Sigma-Aldrich). Fluorescent microscopy images were collected using a Plan Apo VC 60x/1.40 objective with immersion oil in a Nikon Eclipse Ti microscope using 387 nm/447 nm and 472 nm/520 nm filter sets.

### Cell Death Measurements

The release of lactate dehydrogenase (LDH) was measured using the Cytotoxicity Detection kit (Roche) following the manufacturer’s instructions, and expressed as percent of total cell LDH content. This determination was used to measure macrophage cell death after 30 min of incubation with ATP in the presence or absence of different inhibitors followed by 2 h of culture, the same conditions where we found the reduction of MHC I. The determination of cell death after 30 min of incubation with ATP followed by 16 h of macrophage culture was determined as the percentage of propidium iodide (PI, 250 ng for 5 min) positive cells per well. PI fluorescence was captured using a CFI Plan Fluor DLL 10×/0.30 objective in a Nikon Eclipse Ti microscope using 543 nm/593 nm filter set.

### Quantitative Reverse Transcriptase–polymerase Chain Reaction (RT-PCR) Analysis

BMDM in 12 well plates were incubated 30 min in the presence or absence of ATP (5 mM), then washed with PBS, followed by incubation for 2 h with 1 µg/ml LPS or vehicle. After LPS stimulation, total RNA was extracted using the RNeasy Mini kit (Qiagen), followed by reverse transcription using iScript cDNA Synthesis (Bio-Rad) with oligo(dT). The mix SYBR Premix ExTaq (Takara) was used for quantitative PCR in iCycler MyiQ thermocycler (Bio-Rad). Specific primers for quantitative PCR were purchased from Qiagen (QuantiTech Primer Assays). Relative gene expression levels were calculated using the 2^−ΔΔCt^ method normalized to GAPDH expression level for each treatment and the fold increase in gene expression was relative to resting (non LPS) condition.

### Statistical Analysis

All data are shown as mean values and error bars represent standard error (s.e.m.) from the number of assays indicated in the figure legends. The comparisons among the groups were made using one-way analysis of variance and Bonferroni’s test using Prism software (Graph-Pad Software, Inc.). *p* value was considered significant if p<0.05; p>0.05 not significant (ns).

## Results

### P2X7R Activation Induces a Decrease of Surface MHC I Protein

**Figure 1:**
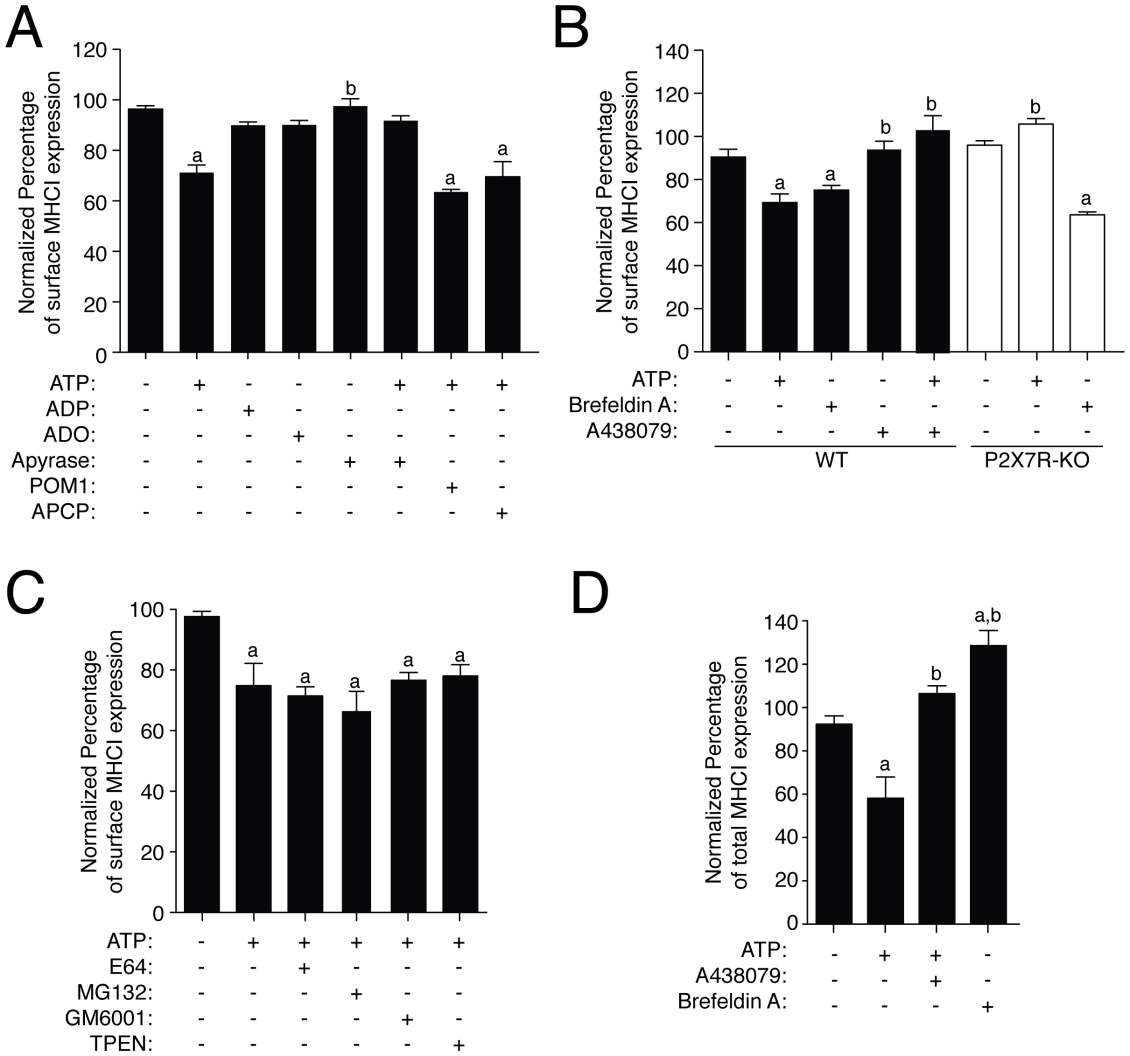
Expression of MHC I is decreased by P2X7R activation. (A) BMDM were transiently incubated during 30 min with ATP (5 mM), ADP (0.1 mM), ADO (0.1 mM), POM1 (0.1 mM), APCP (10 µM), apyrease (3 U/ml) and further incubated 2 h without stimulation in physiological buffer before staining for surface MHC I molecules. (B) BMDM from WT (black bars) or P2X7R deficient mice (white bars) were transiently treated with ATP (5 mM, 30 min) in the presence or absence of specific P2X7R antagonist A438079 (50 µM) and further incubated 2 h without ATP in physiological buffer or incubated with 5 µg/ml of Brefeldin A during 180 min before staining for surface MHC I. (C) BMDM were transiently incubated with ATP (5 mM, 30 min) in the presence of the protease inhibitor E64 (100 µM), the proteasome inhibitor MG132 (50 µM), the broad-range metalloproteinase inhibitor GM6001 (0.5 µM) or the zinc chelator TPEN (50 µM) added 10 min before and during ATP application, then the cells were further incubated 2 h without stimulation in physiological buffer before staining for surface MHC I. (D) Total (intracellular and surface) MHC I detection from BMDM treated as in (B) was measured by cell permeabilization. In all panels cells were stained with APC-conjugated anti-MHC I antibody on ice for 30 min and then analyzed by flow cytometry (*n = *4–9 independent experiments). Percentages of normalized mean intensity fluorescence (MFI) values were calculated respect to untreated cells; *a* denotes *p*<0.05 *vs* resting condition; *b* denotes *p*<0.05 *vs* ATP treated cells.

We found that ATP treatment of macrophages resulted in a significant decrease of the mean fluorescent intensity (MFI) of extracellular membrane MHC I staining measured by flow cytometry (reduction of 21.16±5.432%, *n = *9; Fig. 1A). This decrease was reverted when ATP was degraded by apyrase treatment and appears specific for ATP nucleotide, since it was not observed when macrophages were incubated with ADP or adenosine (Fig. 1A). Furthermore, the inhibition of NTPDase with POM1 or 5′-ectonucleotidase using APCP did not impair the decrease of MHC I induced by ATP (Fig. 1A). All these evidences suggest that ATP was not acting after degradation in the form of ADP or adenosine. This effect appears dependent on P2X7R activation since it was abolished when the specific P2X7R antagonist A438079 was incubated together with ATP or when ATP was applied to macrophages derived from P2X7R deficient mouse (Fig. 1B). As a control, brefeldin A, which impairs delivery of MHC I molecules from the ER to the plasma membrane, was able to induce a decrease of membrane MHC I in both WT and P2X7R deficient macrophages (reduction of 15.36±4.173% and 32.25±3.247% in WT and P2X7-KO respectively, *n = *8, Fig. 1B).

As shown in Fig. 1C, the decrease of extracellular MHC I appears independent on metalloproteinase activity, as the broad-spectrum metalloproteinase inhibitor GM6001 and the Zn^2+^-chelator TPEN did not restore surface MHC I. Similarly, inhibition of lysosome protein degradation by using a broad cathepsin cysteine protease inhibitor (E64) or proteasome blocking by using MG132 did not affect surface MHC I reduction caused by ATP stimulation (Fig. 1C).

We then measured total cellular MHC I content by permeabilizing the cell membrane to explore if P2X7R was inducing an intracellular accumulation of MHC I. ATP treatment also resulted in a reduction of total MHC I, whereas brefeldin A treated cells presented significantly higher amounts of total MHC I (Fig. 1D). Specific P2X7R antagonist A438079 inhibited ATP induced reduction of total MHC I (Fig. 1D).

### Extracellular ATP Impairs MHC I Peptide Presentation in APCs through P2X7R Activation

**Figure 2:**
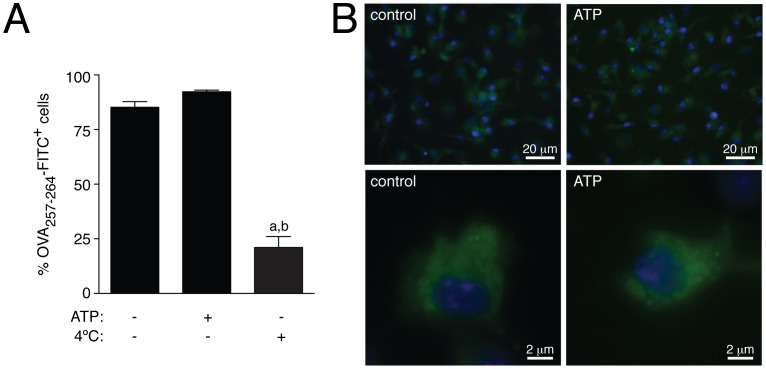
Activation of P2X7R did not affect peptide uptake into macrophages. (A) BMDM treated or not with ATP (30 min, 5 mM) were further incubated 45 min with OVA_257–264_-FITC in presence of 5 µg/ml brefeldin A and then analyzed for peptide uptake by flow cytometry. Percentages of number of FITC positive cells are represented. Peptide incubation at 4°C was carried out as positive control; *n = *6 independent experiments. (B) BDMD treated as in A were examined by fluorescent microscopy. Representative pictures are shown from three independent experiments; *a* denotes *p*<0.05 *vs* resting condition; *b* denotes *p*<0.05 *vs* ATP treated cells.

We next asked whether the presence of extracellular ATP could also induce a reduction of extracellular MHC I-oligopeptide complexes presentation. We first demonstrated that P2X7R stimulation did not affect antigen peptide uptake. Antigen uptake was measured by using FITC label OVA_257–264_ peptide. As shown in Figure 2A, the number of OVA_257–264_-FITC positive cells treated or not with extracellular ATP was not different, and was significantly higher compared to control macrophages incubated at 4°C with the peptide to impair active uptake. Fluorescence microscopy also confirmed this result and images showed no difference in peptide loading or localization in cells treated or not with ATP (Fig. 2B).

**Figure 3:**
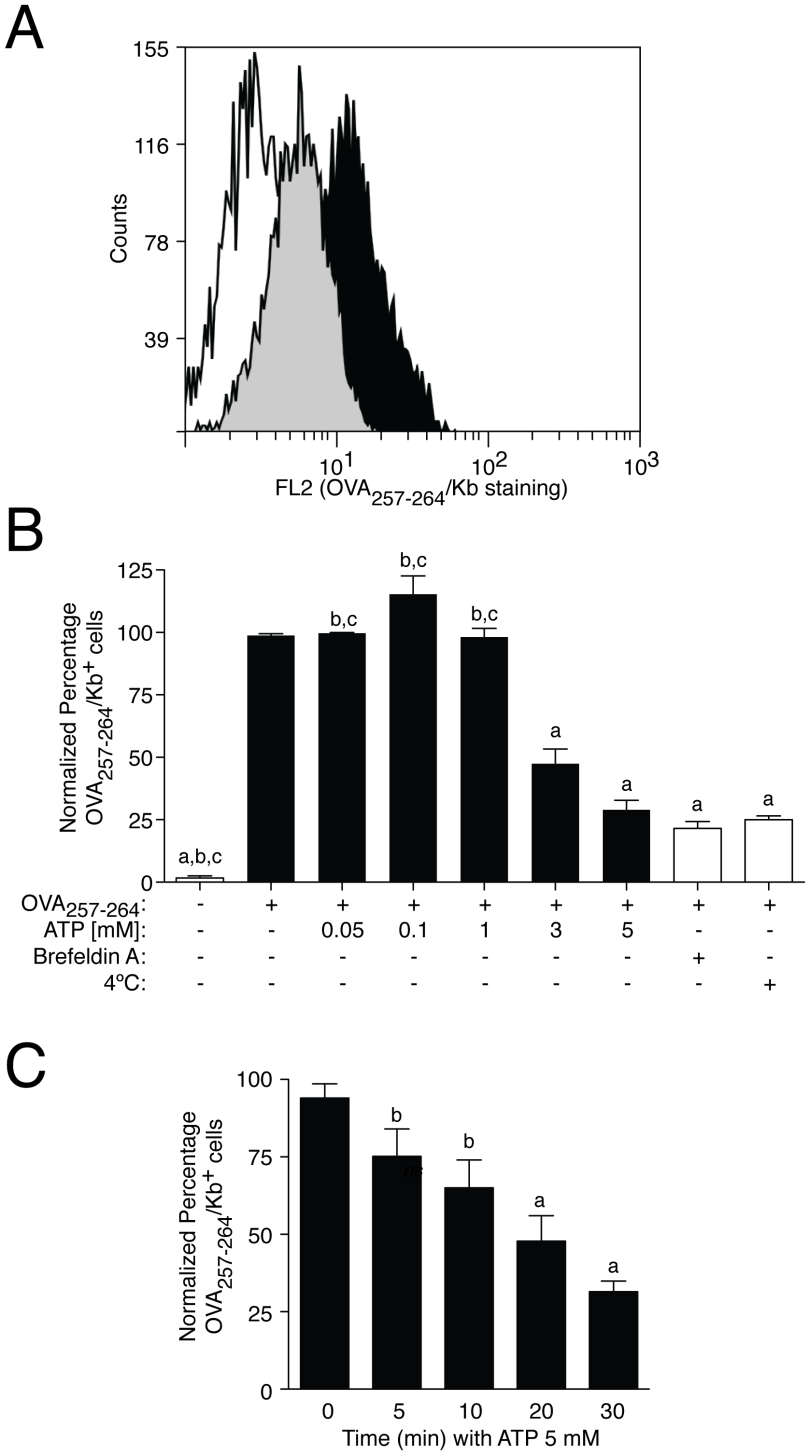
Extracellular ATP-dependent P2X7R activation impairs peptide presentation. (A) Representative flow cytometry of BMDM incubated 2 h with OVA_257–264_ and stained with anti-MHC I(H-2K^b^)pOVA_257–264_-PE conjugated antibody. The white histogram represent no peptide incubation, the grey histogram shows cells treated with ATP 5 mM during 30 min before peptide incubation and the black histogram corresponds with peptide incubation without ATP treatment. (B) BMDM were treated with increasing concentrations of ATP during 30 min before peptide incubation (black bars). Controls for OVA_257–264_ presentation were done by peptide incubation at 4°C or in presence of 5 µg/ml of Brefeldin A or with no OVA_257–264_ (white bars); *n = *3–10 independent experiments; *a* denotes *p*<0.05 *vs* resting condition; *b* denotes *p*<0.05 *vs* 5 mM ATP treated cells; *c* denotes *p*<0.05 *vs* 3 mM ATP treated cells. (C) BMDM were treated with 5 mM ATP during different times, and then subjected to peptide presentation; *n = *3–13 independent experiments; *a* denotes *p*<0.05 *vs* 0 min incubation time; *b* denotes *p*<0.05 *vs* 30 min ATP treatment. In all experiments, after OVA_257–264_ incubation, presentation was analyzed by staining of surface OVA_257–264_/K^b^ complexes. Percentages of normalized number of presenting OVA_257–264_ cells were calculated respect to OVA_257–264_ presentation in the absence of ATP (control conditions) are represented in all graphs.

**Figure 4:**
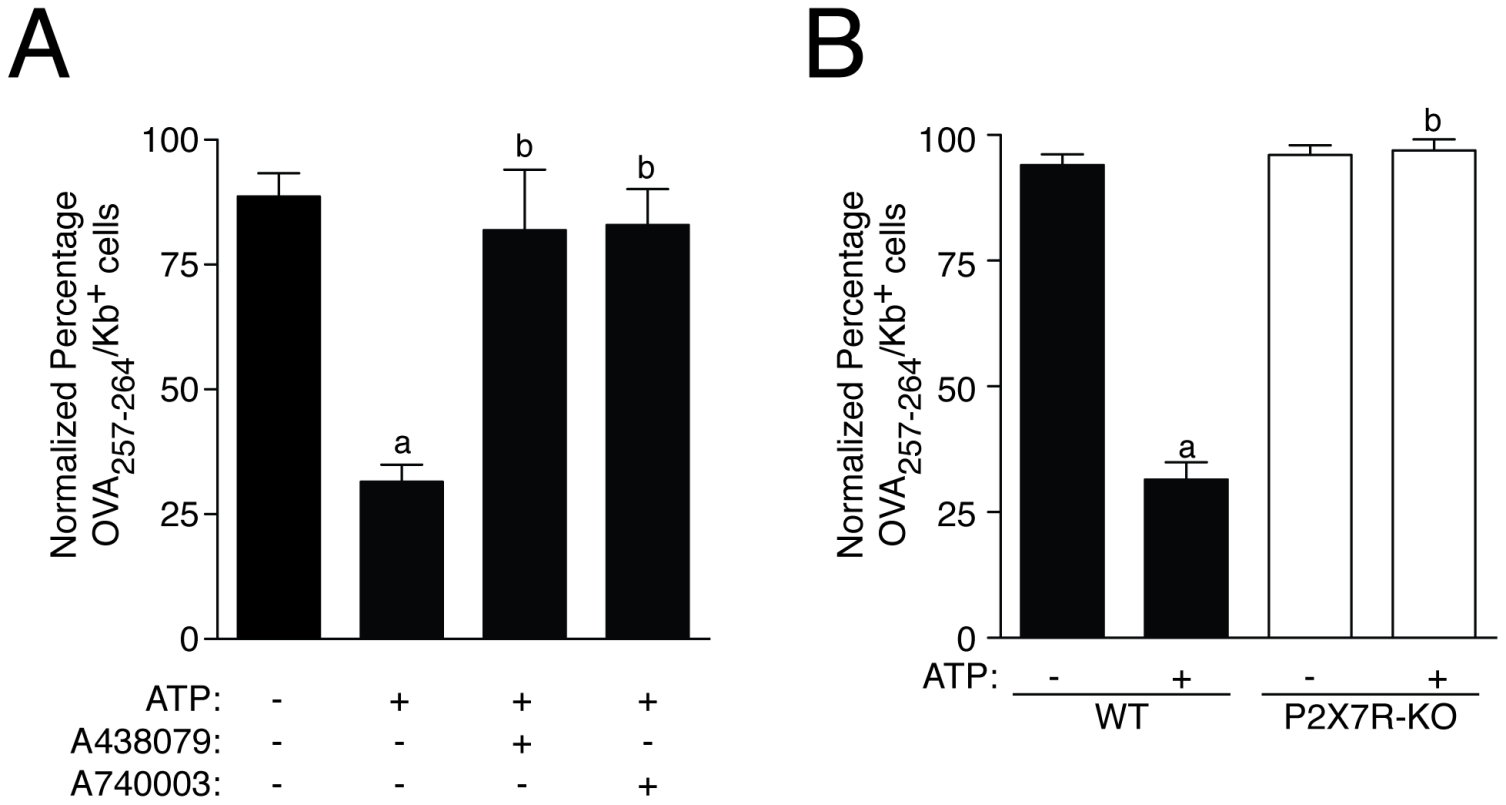
Genetic and pharmacological P2X7R inhibition restores peptide presentation impaired by extracellular ATP. (A) BMDM were treated with 50 µM of specific P2X7-receptor antagonist A438079 or A740003 10 min before and during ATP (5 mM) incubation; *n = *3–4 independent experiments. (E) BMDM from P2X7R deficient mice (P2X7R-KO, white bars) were compared with wild type BMDM (WT, black bars); *n = *4–5 independent experiments Percentages of normalized number of presenting OVA_257–264_ cells were calculated respect to OVA_257–264_ presentation in the absence of ATP (control conditions) are represented in all graphs; *a* denotes *p*<0.05 *vs* resting condition; *b* denotes *p*<0.05 *vs* ATP treated cells.

However, detection of the complex MHC I:OVA_257–264_ by flow cytometry, demonstrate that 5 mM ATP reduced OVA_257–264_ peptide presentation through MHC I to ∼ 65% (Fig. 3A and B). This reduction was higher than the reduction of total or surface MHC I induced by the same concentration of ATP. Extracellular ATP inhibited OVA_257–264_ presentation in a concentration and time dependent manner, being statistically significant with concentrations of extracellular ATP higher than 3 mM and after 20 min of stimulation (51.32±5.98% and 46.22±4.39% of inhibition respectively, *n = *3 for both conditions) (Fig. 3B and C). Blocking peptide presentation by using brefeldin A or by incubation of the cells at 4°C during peptide loading, which inhibit active pinocytosis, suggest that OVA_257–264_ was following the classical peptide presentation pathway in our experimental conditions (Fig. 3B). MHC I peptide presentation was restored by using P2X7R antagonists A438079 or A740003 (Fig. 4A), or by using macrophages derived from P2X7R deficient mice (Fig. 4B).

### Inhibition of MHC I Peptide Presentation is Independent on Cell Death or Intracellular Calcium Signaling

**Figure 5:**
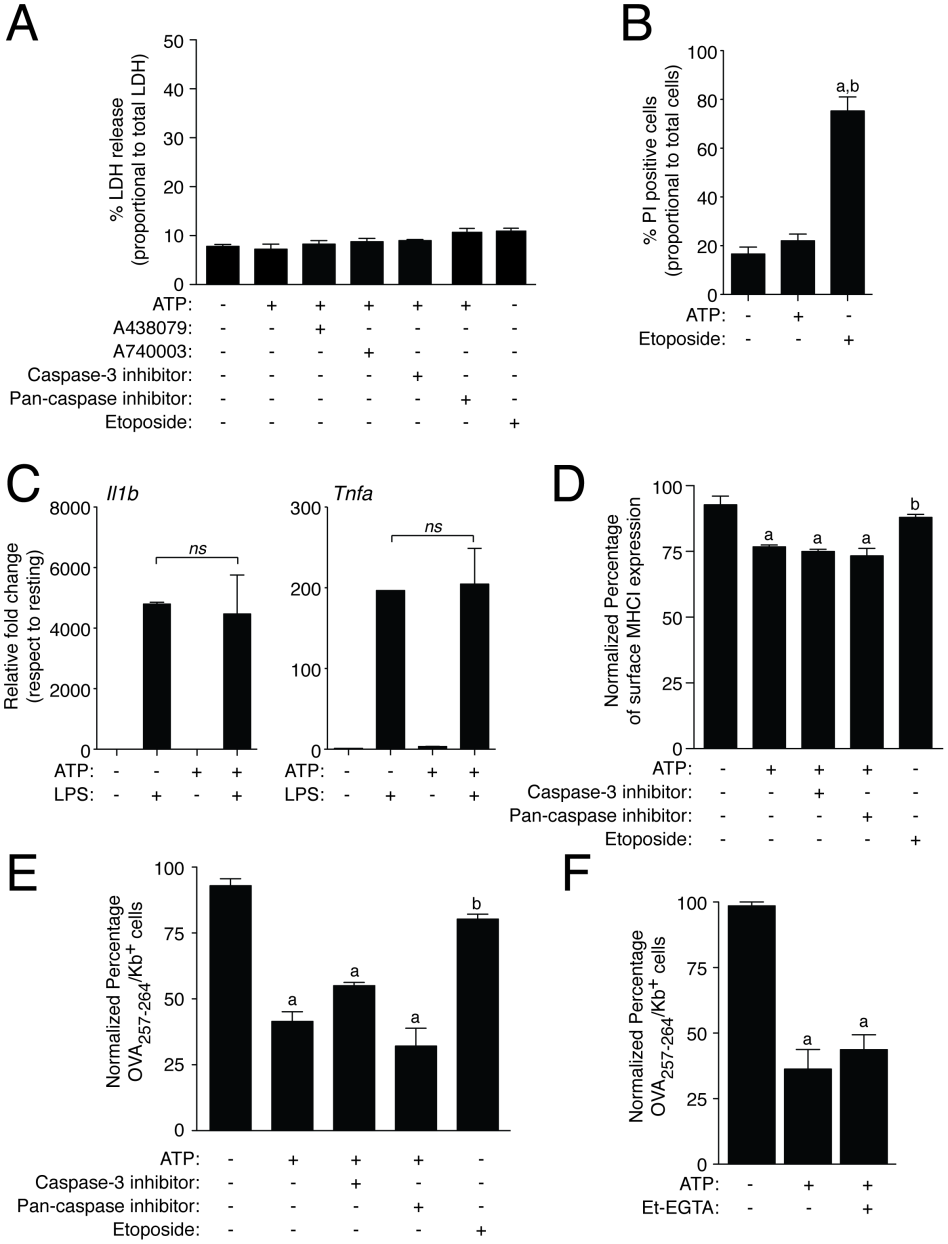
Cell death and intracellular Ca^+2^ are not implicated in ATP-dependent impaired presentation. (A) Presence of LDH in BMDM culture medium from resting conditions or incubated with ATP (5 mM, 30 min) or with the apoptotic inducer etoposide (10 µg/ml) and treated or not with 50 µM of specific P2X7R antagonists (A438079 or A740003), 50 µM of the pan-caspase inhibitor (Q-VD-OPh) or 50 µM of the caspase-3 inhibitor (Ac-DEVD-CHO). Results are expressed as percentage of LDH activity respect to total cellular LDH activity; *n = *3–7 independent experiments. (B) Percentage of propidium iodide (PI) positive macrophages after stimulation with a transient ATP application (5 mM, 30 min) and further culture for 16 h without ATP or incubated with etoposide (10 µg/ml) for 12 h; *n = *3 independent experiments. (C) Relative gene expression for *Il1b* and *Tnfa* measured by qPCR in macrophages treated with a transient ATP stimulation (5 mM, 30 min) and then further activated with LPS (1 µg/ml) for 2 h. Data is normalized to GAPDH and presented as relative values to LPS untreated cells; *n = *3 independent experiments. (D) BMDM from WT mice were incubated with etoposide (10 µg/ml) for 2 h or with a transient ATP stimulation (5 mM, 30 min) in presence or absence of the pan-caspase inhibitor Q-VD-OPh (50 µM) or the caspase-3 inhibitor Ac-DEVD-CHO (50 µM), washed and further incubated for 2 h without ATP stimulation but with the presence of caspase inhibitors. Cells were stained with APC-conjugated anti-MHC I antibody and then analyzed by flow cytometry, *n = *4 independent experiments. Percentages of normalized mean intensity fluorescence (MFI) values shown were calculated respect to untreated cells. (E–F) Staining of surface OVA_257–264_/K^b^ complexes in resting BMDM or incubated with etoposide (10 µg/ml) for 2 h or with a transient ATP stimulation (5 mM, 30 min) and treated or not with 50 µM of pan-caspase inhibitor (Q-VD-OPh), 50 µM of caspase-3 inhibitor (Ac-DEVD-CHO) (E) or with calcium free buffer (Et-EGTA, F); *n = *3–5 independent experiments for E-F. Percentages of normalized number of presenting OVA_257–264_ cells were calculated respect to OVA_257–264_ presentation in the absence of ATP (control conditions); *a* denotes *p*<0.05 *vs* resting condition; *b* denotes *p*<0.05 *vs* ATP treated cells.

We next determined whether the inhibition of MHC I peptide presentation induced by extracellular ATP was mediated directly by cell death induced after P2X7R activation. As a marker of cell death we measured LDH release into the extracellular medium of macrophages treated with a transient ATP stimulation (30 min, 5 mM) followed by 2 h incubation time without ATP to mimic conditions where cellular MHC I was reduced. We found similar levels of extracellular LDH in macrophages treated or not with ATP, and in the presence or absence of specific P2X7R antagonists (LDH levels <10% in all situations, Fig. 5A). Furthermore, to investigate if apoptosis cell death was involved in the inhibition of the antigen presentation, we treated macrophages with a general pan-caspase or specific caspase-3 inhibitor before and during ATP stimulation or with the apoptotic inducer etoposide. Apoptosis inhibition by targeting caspases could not restore the effect of extracellular ATP in MHC I reduction or OVA peptide presentation and did not alter levels of extracellular LDH (Fig. 5A, D and E). When macrophages were cultured for 16 h after the transient ATP treatment (5 mM, 30 min), cell death was not increased when compared with untreated cells and as control the apoptosis inducer etoposide significantly raised cell death (Fig. 5B). Additionally, after 30 min of ATP stimulation macrophages were functional and LPS treatment induced similar expression of *Il1b* and *Tnfa* in macrophages pre-treated or not with ATP (Fig. 5C). Apoptosis induced by etoposide fails to decrease expression levels of surface MHC I and OVA peptide presentation (Fig. 5D and E), suggesting that the reduction of peptide presentation via MHC I observed after ATP treatment was not a consequence of P2X7R induced cell death.

Intracellular calcium rise is a well-known effect of ATP-dependent P2X7R activation implicated in receptor downstream signaling. However, when we use a buffer free of calcium (Et-EGTA) to impair intracellular calcium rise upon P2X7R stimulation, extracellular ATP application in BMDM had no effect on the reduction of peptide presentation (Fig. 5F).

### ATP-dependent P2X7R Activation Impairs CD8^+^ T cells Activation by APCs

**Figure 6:**
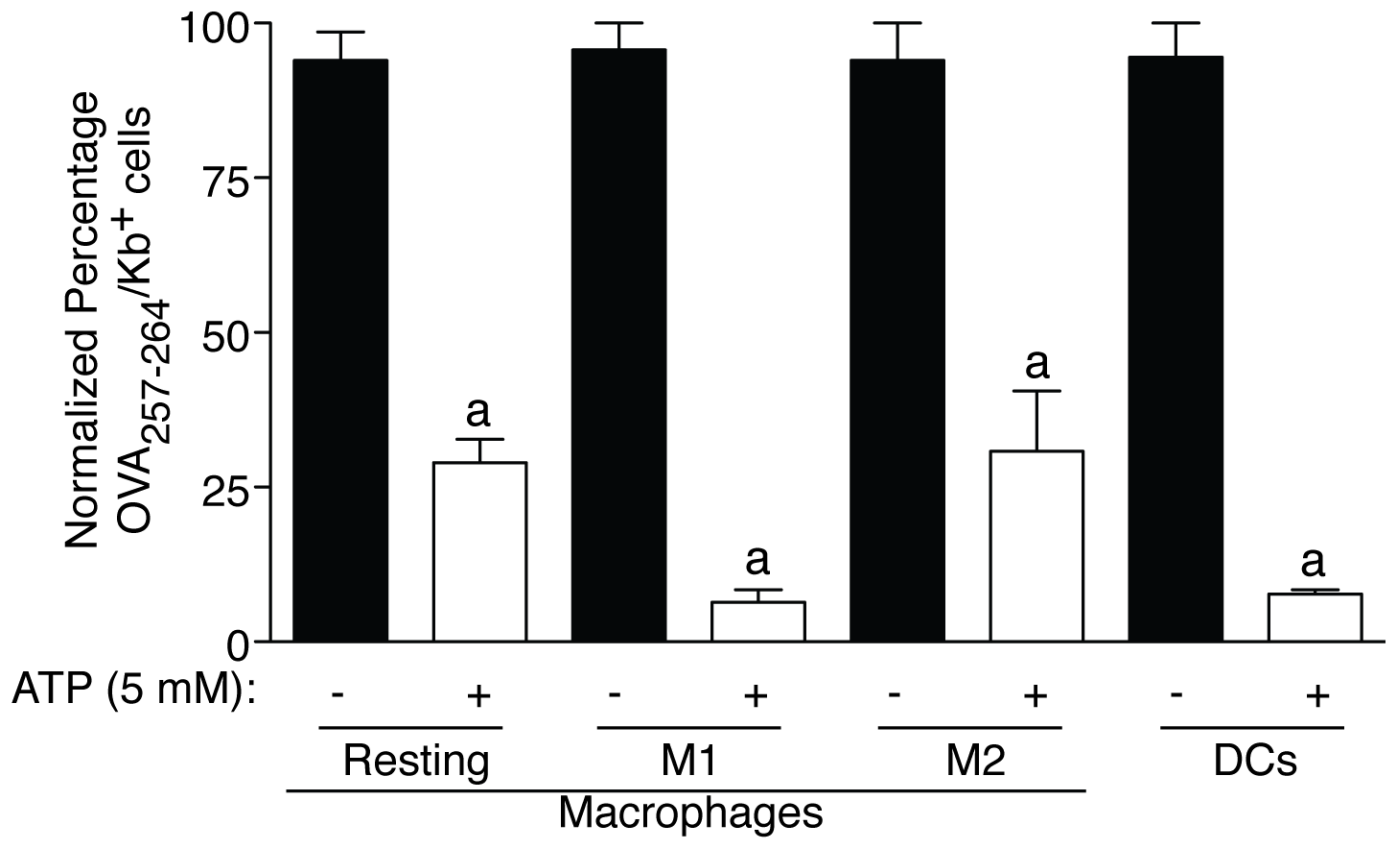
Inhibition of presentation induced by P2X7R activation is found in different macrophages states and DCs. Resting macrophages, classically activated (M1) macrophages with 1 µg/ml LPS and 20 ng/ml IFNγ for 4 h, alternatively activated (M2) macrophages with 20 ng/ml IL-4 for 4 h and DCs were assayed for presentation after being treated (white bar) or not (black bar) with 5 mM ATP for 30 min; *n = *3–10 independent experiments. Percentages of normalized number of presenting OVA_257–264_ cells are represented in all graphs as assessed by staining of surface OVA_257–264_/K^b^ complexes with anti-MHC I(H-2K^b^)pOVA_257–264_-PE conjugated antibody and were calculated to the maximum value of OVA_257–264_ presentation in the absence of ATP (control conditions); *a* denotes *p*<0.05 *vs* resting condition of each macrophage state.

Extracellular ATP was able to impair presentation in both macrophages and DCs and was effective among different macrophage activation states: resting, classical (M1) and alternative (M2) activated macrophages (Fig. 6). The inhibition of antigen presentation in M2 macrophages was similar to resting conditions (63.23±11.27% and 65.04±7.424 respectively, *n = *3 for both conditions), meanwhile in M1-polarized macrophages and in DCs the inhibition was near 90% (89.3±4.79% and 86.97±3.205% respectively, *n = *3 for both conditions) (Fig. 6).

**Figure 7:**
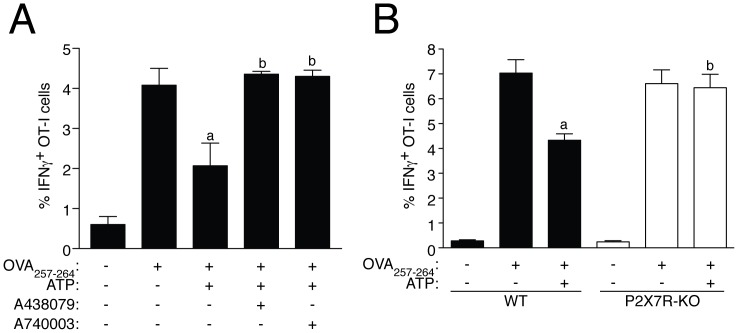
P2X7R activation in BDMD impairs activation of CD8^+^ T cells.

We further determined whether P2X7R activation could also result in a decrease of CD8^+^ T cells activation through MHC I:OVA_257–264_. We used splenocytes from OVA-specific TCR-transgenic OT-I mice and the percentage of OT-I cells producing IFN-γ after co-culture with macrophages presenting OVA_257–264_ was significant reduced when macrophages were pre-treated with ATP (Fig. 7A). This reduction was abolished when a specific P2X7R antagonist was used together ATP (Fig. 7A) or when we applied ATP to macrophages derived from mice deficient on P2X7R (Fig. 7B).

## Discussion

In this study, we examined the role of P2X7R in the activation of CD8^+^ T cells through MHC I complex. We found that P2X7R stimulation in APCs reduces cellular and surface MHC I levels, with the consequence to impair peptide presentation and activation of CD8^+^ T cells through MHC class I molecules. Selective blocking or deficiency on P2X7R restored CD8^+^ T cell activation in response to extracellular ATP treatment. P2X7R activation did not impair peptide uptake or macrophage viability, but resulted in a reduction of membrane MHC I complexes.

High concentrations of extracellular ATP (in the range of mM) are required to activate P2X7R, this being a unique property of the P2X7R [25], which has been often used to question the functionality of P2X7R *in vivo*, where it is thought that extracellular ATP levels are below the threshold to activate P2X7R. However, recent studies have found high levels of extracellular ATP associated with damaged and inflamed areas *in vivo* [10], [12], [13] and P2X7R itself acting as a pathway for ATP release [26], supporting the idea for P2X7R serving as a positive feedback loop amplifying purinergic signaling *in vitro* and *in vivo*. In our study, high concentrations of extracellular ATP promote a decrease on extracellular membrane MHC I protein, without apparent retention in intracellular vesicles, as total cellular MHC I presented the same reduction. This reduction was independent on ATP degradation by ectonucleotidases and subsequent ADP or adenosine signaling, and was found to be specific on P2X7R activation. MHC I reduction induced by P2X7R activation results in the inhibition of antigen presentation through MHC I class complex and the impairment of the activation of CD8^+^ T cells, therefore this mechanism appears as a novel repressor of adaptive CD8^+^ T cell immunity. In this study we assessed the percentage of IFNγ^+^ cells by flow cytometry from OVA-specific TCR-transgenic OT-I mice to determine specific CD8^+^ T cell stimulation. In accordance with other studies [27], [28], we found that this population was induced after stimulation with the specific OVA antigen presented through MHC I class molecules on APCs. It is known that infection of APCs with some pathogens such as *Mycobacterium tuberculosis*, influenza A virus or the Japanese encephalitis virus are able to impair CD8^+^ T cell activation through MHC I antigen presentation [29], [30], [31]. This mechanism occurs via a reduction of proteasome function that decreases the production of antigenic peptides [29], [30], [31]. In our study we have found another pathway to control CD8^+^ T cell activation through the action of ATP as a ‘sterile’ danger signal in APCs, which involved the reduction of MHC I presenting molecules. In our study P2X7R activation in APCs was able to reduce the activation of CD8^+^ cells to produce IFNγ by ∼ 50%, and this decrease was similar to the reduction of the activation of CD8^+^ cells found after APCs infection [29], [30], [31].

It is known that MHC I at APCs cell surface could undergo shedding from the plasma membrane by enzymatic processing involving Zn^2+^-dependent membrane-bound metalloproteinases [32], [33]. P2X7R can activate different metalloproteinases [34], but the loose of MHC I after P2X7R stimulation did not depend on metalloproteinase activity, since a general metalloproteinase inhibitor or chelation of Zn^2+^ had no effect on the reduction of MHC I molecules. The reduction of MHC I was also independent on macrophage cell death and therefore was not an unspecific consequence of reduced cellular function, which is characteristic of early stages of apoptosis. We found that extracellular LDH levels were unchanged in macrophages treated with ATP and inhibition of caspases did not affect the reduction of MHC I. However, recent studies have found that transient stimulation of P2X7R in murine macrophages leads to a delayed cell death several hours later [35], [36]. In our experimental set up, we could not found signs of delayed macrophage cell death after a transient ATP application, whereas the classical apoptosis inducer etoposide increased macrophage cell death. The differences on cell viability after P2X7R stimulation found in our study could be explained by the use of different types of macrophages, whereas we used mouse bone marrow derived macrophages, other works reporting high rates of macrophage death use primary mouse peritoneal macrophages [35], [36]. Bone marrow derived macrophages present negligible cell death in response to P2X7R activation (5% increase on extracellular LDH after 30 min of 5 mM ATP application), compared to peritoneal macrophages which death level rise close to 80%. Finally, after ATP stimulation, bone marrow derived macrophages were functional, since they were able to increase pro inflammatory gene expression in response to LPS treatment. Therefore, the reduction of MHC I molecules induced by P2X7R activation appears independent on early events of cell death. This idea is further supported by the fact that the apoptosis inducer etoposide did not decrease levels of MHC I or antigen presentation at early times.

Recycled MHC I is usually degraded via the proteasome and under certain circumstances via the lysosomal pathway [9], [37], [38], and since P2X7R stimulation causes lysosomal desestabiliziation [15], we next discarded that MHC I reduction induced by P2X7R stimulation was due to intracellular degradation, as the lysosome cysteine protease inhibitor E64 or the proteasome inhibitor MG132 had no effect on MHC I levels after ATP treatment. Therefore, we could hypothesize that MHC I is being released following P2X7R activation through an exosome shedding, since it is known that P2X7R induce the release of exosome containing MHC II protein [17]. Although, it is tempting to speculate that in our experimental setup MHC I could follow a microvesicle release pathway, rather than an exosome release, since we found the reduction of MHC I to be independent on intracellular Ca^2+^ rise, and whereas exosomes release is dependent on intracellular Ca^2+^ [39], [40], microvesicle release is a Ca^2+^-independent process [15], [41].

Effective cross-presentation though MHC I molecules has been very successful in eliminating tumors in mouse model systems, but this success has not yet translated to human cancer patients. Tumor antigens are usually not presented by tumor MHC I molecules and needs to be cross-presented by DCs in order to achieve a strong CTL response against such antigens [42]. Tumor malignant cells also lack MHC I surface expression to escape immune system surveillance [43]. This means that the DC acquires the antigen from the tumor cell and cross-presents it by its own MHC I molecules to activate the corresponding tumor-specific CTLs [42], [44], [45]. Moreover, tumors present an immunosuppressive environment by the local secretion of inhibitory factors such as IL-10, which inhibit APCs activation and cross-presentation [46], [47]. In fact, tumor associated macrophages (TAMs) are in an alternative activation state (the so called M2 macrophages), which displays a reduced pro-inflammatory phenotype and promote tissue growth [48], [49]. It has been also found that in the core of solid tumors there are high levels of extracellular ATP [50]. However, such ATP will not promote pro-inflammatory cascades in the tumor APCs, since ATP acting on M2 macrophages is an anti-inflammatory signal blocking the inflammasome and NF-κB signaling [24], [51]. In the present work we additionally show that high levels of ATP are able to impair MHC I peptide presentation in both types of activated macrophages, the M1 and M2. This mechanism could serve as a novel pathway for tumor evading immune response by inhibiting tumor peptide presentation via P2X7R activation in TAMs. Furthermore, extracellular ATP in the tumor environment will also promote tumor growth and invasion by activating P2X7R on cancer cells [52], [53], [54], and we can speculate that in tumor cells P2X7R could also reduce the levels of MHC I to evade the immune system. So overall, in the tumor microenvironment, an anti-P2X7R therapy could represent a novel and effective treatment to (*i*) reduce tumor growth/invasion and (*ii*) increase associated tumor antigen presentation by APCs-MHC I and promote an endogenous tumor specific CTL response.

In conclusion, P2X7R activation is a novel route to impair MHC I activation of CD8 T cells which could be important in anti-tumor immune response.
